# Common genetic variation associated with adult subcortical brain volume is also associated with subcortical brain volume at birth

**DOI:** 10.3389/fnins.2025.1546845

**Published:** 2025-06-10

**Authors:** Harriet Cullen, Konstantina Dimitrakopoulou, Hamel Patel, Charles Curtis, Dafnis Batalle, Oliver Gale-Grant, Lucilio Cordero-Grande, Anthony Price, Joseph V. Hajnal, A. David Edwards

**Affiliations:** ^1^School of Biomedical Engineering and Imaging Sciences, Centre for the Developing Brain, King's College London, London, United Kingdom; ^2^Department of Medical and Molecular Genetics, School of Basic and Medical Biosciences, King's College London, London, United Kingdom; ^3^Translational Bioinformatics Platform, NIHR Biomedical Research Centre, Guy's and St Thomas' NHS Foundation Trust and King's College London, London, United Kingdom; ^4^NIHR BioResource Centre Maudsley, NIHR Maudsley Biomedical Research Centre, King's College London, London, United Kingdom; ^5^Department of Forensic and Neurodevelopmental Sciences, Institute of Psychiatry, Psychology & Neuroscience, King's College London, London, United Kingdom; ^6^Biomedical Image Technologies, ETSI Telecomunicación, Universidad Politécnica de Madrid & CIBER-BBN, ISCIII, Madrid, Spain

**Keywords:** neonate, neuroimaging, MRI, subcortical brain volume, polygenic risk score, single nucleotide polymorphism (SNP)

## Abstract

**Introduction:**

Recent genome-wide association studies have identified numerous single nucleotide polymorphisms (SNPs) associated with subcortical brain volumes. These studies have been undertaken in largely adult cohorts. In this work we explore the role of common genetic variability in fetal and perinatal brain development. We investigate how genetic variation, known to be associated with adult subcortical brain volume, affects the infant subcortical brain.

**Methods:**

We examine the influence of specific genetic loci and genome-wide polygenic scores on development of the fetal brain. Using a cohort of 208 term-born infants from the Developing Human Connectome Project, we ask whether eight SNPs, previously associated with adult subcortical brain volumes, show similar associations at birth. In addition, we compute genome-wide polygenic scores for the amygdala, brainstem, caudate, hippocampus, pallidum, putamen and thalamus and ask whether these scores are associated with the corresponding neonatal brain volumes.

**Results:**

We find that the association between SNP rs945270 and putamen volume, seen in adults, is present at birth (*p* = 3.67 × 10^−3^, β = 0.13, SE = 0.04). We also show that neonatal hippocampal, brainstem, putamen and thalamic volumes are all significantly associated with the genome-wide polygenic scores for their corresponding adult subcortical brain volume.

**Conclusions:**

Our results suggest that common genetic variation, important in shaping adult subcortical brain volume, also plays a significant role in fetal and perinatal brain development.

## 1 Introduction

Imaging-genetic studies have identified many genome-wide significant associations between MRI brain-imaging phenotypes and single nucleotide polymorphisms (Elliott et al., 2018; Hibar et al., 2015; Satizabal et al., 2019). These studies have improved our understanding of how common genetic variation influences both normal and abnormal brain development. However, to-date such studies have been almost exclusively undertaken in adults and adolescents. The extent to which common genetic variation, known to be important in shaping adult brain volume, varies over the life course is still not well understood. Specifically, genetic variation important in influencing brain morphology in early infancy remains largely unexplored. Fetal and early neonatal brain development is the most dynamic phase of human brain development and dysregulation of gene expression during this period likely contributes to risk of neuropsychiatric disease and neurodevelopmental disorders (Silbereis et al., 2016).

The paucity of neonatal imaging-genomic studies is not surprising given the challenges in acquiring neuroimaging in early infancy. However, the result is an important gap in our knowledge regarding genetic variation relevant to early brain development. Studies that have explored the stability of genetic variation impacting brain volume over the life course suggest concordance (Hibar et al., 2015; Lamballais et al., 2021), however, these studies do not include imaging in the perinatal period.

Several recent adult brain-imaging genome-wide association studies (GWAS) have focused on subcortical brain volumes (Hibar et al., 2015; Satizabal et al., 2019; Hibar et al., 2017) identifying single nucleotide polymorphisms (SNPs) important in influencing these structures. Differences in the subcortical brain and its related circuitry are thought to be associated with cognitive function, psychiatric and movement disorders (Shepherd, 2013; Ellison-Wright et al., 2008; Schmaal et al., 2016; Van Erp et al., 2016). The subcortical brain is also particularly vulnerable to abnormal development in infants born preterm (Boardman et al., 2006; Srinivasan et al., 2007), abnormalities which are associated with adverse neurocognitive outcomes (Inder et al., 2005; Boardman et al., 2010).

This study starts to address the knowledge gap that exists regarding genetic variation relevant to early brain development and asks if common genetic variation, important in shaping adult brain volumes, is also associated with brain volume at birth. We focus on subcortical structures where robust associations have already been established in adults (Satizabal et al., 2019). Using data from the Developing Human Connectome Project (http://www.developingconnectome.org) (Edwards et al., 2022) which provides high quality neonatal brain imaging and genome-wide SNP data, we ask if key subcortical SNP-volume associations, present in adults, exist at birth.

Variability in subcortical brain volume is moderately to highly heritable and this is in part explained by the cumulative effects of common genetic variation throughout the genome (Satizabal et al., 2019). To complement our work looking at single SNP-volume associations we calculate genome-wide polygenic scores (GPSs) for each neonate, for each subcortical region, using summary statistics from the adult subcortical GWAS (Satizabal et al., 2019). We then ask if these scores are associated with the corresponding neonatal brain volume.

GPSs provide an estimate of an individual's genetic liability for a particular trait or phenotype. They are calculated by summing an individual's risk alleles genome-wide, weighted by the allele's effect size, which is estimated from the GWAS for the relevant trait or phenotype, in this case subcortical volume. GPSs are not limited to SNPs reaching genome-wide significance and therefore capture a broader range of genetic variation relevant to the trait or phenotype in question.

Recently, Lamballais et al. (2021) observed a significant association between GPSs for adult subcortical brain volumes and the corresponding brain volumes in children aged 10. Our work extends the MRI-GPS exploration to earlier in the life-course, asking if GPSs for adult subcortical brain volumes are also associated with the corresponding brain volumes at birth.

## 2 Methods

### 2.1 Sample characteristics

Data for this study were obtained as part of the Developing Human Connectome Project (dHCP) (http://www.developingconnectome.org/). The dHCP was conducted according to the principles of the Declaration of Helsinki. The project has received UK NHS research ethics committee approval (14/LO/1169, IRAS 138070) and written informed consent was obtained from parents.

This study includes infants with genetic data genotyped in June 2019 as part of the first phase of the dHCP genetic analysis, conducted by the NIHR BioResource Center Maudsley Genomics & Biomarker Core Facility. A total of 628 samples were processed. Quality control procedures excluded 25 samples due to low call rate, duplicates, sex mismatches and heterozygosity outliers, and a further 41 were removed due to relatedness, leaving 562 samples. From these, only samples of infants born at term were retained (432 samples). Of these, 422 had accompanying imaging data. One sample was excluded due to marked cerebral pathology in the subcortical brain, leaving 421 samples for subsequent analysis. Three ancestral cohorts were then identified: the full mixed ancestral cohort (*n* = 418) and two further cohorts that were subsets of this larger cohort. These were a European-South Asian cohort (*n* = 258) that contained all individuals of either European or South Asian ancestry and a European cohort (*n* = 208), containing only individuals of European Ancestry, which formed the principal cohort of investigation. A flowchart detailing the selection of these cohorts is shown in Supplementary Figure 1.

Infants were typically scanned within the first few weeks of life (range: 0 weeks to 5 weeks and 6 days). Our study therefore captures the effects of brain development predominantly during the fetal period but also includes a brief perinatal period, dependent on the precise timing of the infant's scan. Sample characteristics for the full mixed-ancestry cohort, the European-South Asian ancestry cohort, and the European ancestry cohort are detailed in Table 1.

**Table 1:** Summary statistics for three neonatal cohorts.

	**European-ancestry cohort**	**European – South Asian ancestry cohort**	**Full mixed-ancestry cohort**
Number of Infants (Male, Female)	208 (110, 98)	258 (139,119)	418 (228,190)
Mean Gestational Age at Birth (weeks)	40.1 ± 1.2	40.1 ± 1.3	40.0 ± 1.2
Mean Postmenstrual Age at Scan (weeks)	41.5 ± 1.7	41.5 ± 1.7	41.2 ± 1.7
Mean Intracranial Volume (cm^3^)	456 ± 62	452 ± 61	444 ± 60

Values indicated are mean and standard deviation.

### 2.2 Imaging data

All subjects underwent Magnetic Resonance Imaging (MRI) scanning at the Evelina Newborn Imaging Center, St Thomas' Hospital, London, UK. T2-weighted MRI brain images were acquired on a 3T Philips Achieva scanner during natural sleep using a dedicated neonatal brain imaging system (Hughes et al., 2017). Hearing protection and physiological monitoring were applied prior to scanning and all scans were supervised by a neonatal nurse and/or pediatrician.

T2-weighted images were used in these analyses and were obtained using a turbo spin echo (TSE) sequence using parameters: TR = 12 s, TE = 156 ms with overlapping slices (resolution 0.8 × 0.8 × 1.6 mm^3^). Motion-correction (Cordero-Grande et al., 2018) and slice-to-volume image reconstruction (Kuklisova-Murgasova et al., 2012) were carried out retrospectively to obtain 0.5 mm^3^ resolution isotropic T2-weighted images. Structural processing followed the pipeline described in Makropoulos et al. (2018): motion- and bias-corrected T2-weighted images were brain extracted and segmented using the Draw-EM neonatal segmentation algorithm based on (Makropoulos et al., 2014).

The segmentation utilizes the atlases manually annotated by Gousias et al. (2012) that divides the brain into 50 regions which were then further subdivided to give a total of 87 regional structures (Makropoulos et al., 2014) including subcortical brain volumes. Estimates of the bilateral volume of the hippocampus, amygdala, caudate, thalamus and brainstem were obtained from this dHCP neonatal structural pipeline (Makropoulos et al., 2018).

Volume estimates for the putamen and pallidum, which were not available as part of the dHCP structural pipeline, were extracted using a neonatal version (Shi et al., 2011) of the Automated Anatomical Labeling (AAL) atlas adapted to a high-resolution dHCP template (Schuh et al., 2018). AAL parcellation propagation was performed as previously described in Taoudi-Benchekroun et al. (2022).

### 2.3 Genetics data

Infant saliva was collected using the Oragene DNA OG-250 kit, DNA was extracted and genotyped for SNPs genome-wide on the Illumina Infinium Omni5-4 v1.2 array. Basic quality control was performed according to the pipeline in Patel et al. (2022). Further quality control was then undertaken, this included the removal of duplicate samples and the removal of individuals with discordant sex information or individuals with a genotyping failure of more than 1% of SNPs.

SNPs were excluded that had a minor allele frequency < = 0.05, were missing in > 1% of individuals or deviated from Hardy-Weinberg equilibrium with a *P* value < 1 × 10^−5^. Non-autosomal SNPs were removed. Further, individuals were excluded if there was genetic evidence of relatedness (pi_hat >= 0.1875). In such cases one member of each pair was randomly retained.

The dataset was imputed to the Haplotype Reference Consortium reference panel (McCarthy et al., 2016) on the Michigan Imputation Server. The VCF files returned were converted to plink files using a genotype calling threshold of 0.9. The imputed markers underwent a second stage of quality control. SNPs were excluded that had a minor allele frequency < = 0.05, were missing in more than 1% of individuals, deviated from Hardy-Weinberg equilibrium with a *P* value < 1 × 10^−5^ or had an imputation Rsq value of less than or equal to 0.8. All quality control was performed with PLINK 1.9 (https://www.cog-genomics.org/plink2).

Ancestry sub-populations were identified by merging our cohort with 2,504 individuals from the 1,000 Genomes Project using a subset of common autosomal SNPs (Auton et al., 2015). These data were then pruned, and we performed a principal-component (PC) analysis in plink and examined sequential PC plots utilizing population labels provided for individuals in the 1,000 Genomes Project to visually identify subpopulations within our sample. Plots of the first two ancestry principal components for our cohort merged with the 1,000 Genomes Project are shown in Supplementary Figure 3.

### 2.4 Single SNP-volume analysis

#### 2.4.1 Selection of SNP-volume pairs

The starting point for this work were the results of Satizabal et al. (2019) who explored SNP-volume associations for the accumbens, amygdala, brainstem, caudate nucleus, globus pallidus, putamen and thalamus using GWAS. Their cohort included almost 40,000 individuals from the Cohorts of Heart and Aging Research in Genomic Epidemiology (CHARGE) consortium, the Enhancing Neuro Imaging Genetics through Meta-Analysis (ENIGMA) consortium and UK Biobank. The hippocampus was not included as it had been recently studied using 33,536 individuals from the CHARGE and ENIGMA consortiums (Hibar et al., 2017).

To identify a set of SNP-volume pairs to explore in our neonatal sample, we selected the most robust association for each subcortical volume from either (3) or (6). For the hippocampus, we included the two most significant associations because of the low minor allele frequency of the most robust association. Details of the eight SNP-volume pairs and their associated *P* values in the original studies (Satizabal et al., 2019; Hibar et al., 2017) are given in Supplementary Table 1. The nucleus accumbens was not included because this volume was not segmented using our neonatal atlases. Of the eight SNP-volume pairs considered, six of the SNPs were intergenic and two were intronic.

#### 2.4.2 Statistical analysis of SNP-volume pairs

We investigated the SNP-volume associations in our neonatal cohort using the same approach as the adult studies in which the associations were identified (Satizabal et al., 2019; Hibar et al., 2017). The additive SNP dosage value was regressed against the bilateral subcortical brain volume of interest. We included gestational age at birth, postmenstrual age at scan, sex, intracranial volume, and ancestry principal components as covariates. Ancestry principal components were specific to the cohort under investigation and were calculated for each of the three cohorts separately. The number of ancestry principal components included was dependent on the cohort under investigation and reflected the ancestral diversity of the cohort under investigation: three, five and nine were used respectively for the European ancestry, European-South Asian ancestry, and full mixed-ancestry cohorts respectively.

The main analysis was undertaken in our European-ancestry cohort (*n* = 208). Eight SNP-volume pairs were explored with a Bonferroni-corrected *P* value for association of *P* < 0.05/8 = 0.00625. To make the best use of the ancestral diversity in our dataset, SNP-volume associations reaching nominal significance in the European-ancestry cohort were also explored in the larger European-South Asian ancestry and the full mixed-ancestry cohorts.

### 2.5 GPSs for adult subcortical brain volumes and neonatal brain volume

The first part of this study explored single genome-wide significant SNP-volume associations, previously established in adults, in our neonatal cohort. We then sought to explore, whether broader genetic variation associated with adult subcortical brain volumes, is also associated with morphological differences in the infant brain. We computed GPSs for seven subcortical brain volumes using summary statistics available from the work of Satizabal et al. (2019) and Hibar et al. (2017). These adult studies were undertaken in individuals of European ancestry, we therefore used our European-ancestry cohort to explore the possible association of adult subcortical GPS with the corresponding neonatal brain volumes.

GPSs are the sum of an individual's risk alleles, weighted by the allele's effect size, which is estimated from the GWAS for the relevant trait or phenotype. GPSs were computed in PRSice-2 (Choi and O'Reilly, 2019) which uses a subset of SNPs, extracted following *P-*value informed clumping, that exceed a specified GWAS *P-*value threshold. Scores were computed for our neonatal cohort at six *P-*value thresholds (1 × 10^−8^, 1 × 10^−6^, 0.001, 0.01, 0.1,1). The 1,000 Genomes project was used as an external linkage disequilibrium reference dataset (Auton et al., 2015).

We used a linear regression model to explore possible association between infant subcortical brain volume and GPS for adult subcortical brain volume, including sex, gestational age at birth, postmenstrual age at scan, intracranial brain volume and three ancestry principal components (computed in our European-ancestry cohort) as covariates. Our principal aim was to assess whether the GPS for a given adult subcortical brain volume is associated with the corresponding neonatal brain volume. Seven subcortical GPSs, computed at six *P-*value thresholds were compared with the corresponding neonatal brain volume. Both the neonatal brain volumes and the differently thresholded GPSs are highly correlated. We used the method proposed by Li et al. (2005) to compute the effective number of independent tests performed, accounting for the correlation structure between measures, giving a corrected *P* value of *P* < 4.16 × 10^−3^. Additional results looking at non-specific associations between all adult subcortical GPSs and all neonatal brain volumes are presented in Supplementary Figure 2.

## 3 Results

### 3.1 Single SNP-volume analysis

Results of the SNP-volume association analysis are detailed in Table 2. There was a statistically significant association between the number of C alleles at SNP rs945270 and putamen volume in our neonatal cohort: a greater number of C alleles was associated with a larger volume (β = 0.128, SE = 0.044, *P* = 3.67 × 10^−3^). Two further SNP-volume pairs had uncorrected *P*-values < 0.05 but did not survive multiple-testing correction: SNP rs61921502 with hippocampal volume (β = −0.106, SE = 0.047, *P* = 0.026) and SNP rs11111090 with brainstem volume (β = −0.072, SE = 0.036, *P* = 0.042). For all of these results the direction of association was the same as in previous adult studies (Satizabal et al., 2019; Hibar et al., 2017). There was no evidence in our neonatal cohort for the SNP-volume associations for the pallidum, caudate, amygdala or thalamus, previously identified in adults.

**Table 2:** SNP-volume results for the European-ancestry (*n* = 208) cohort.

**Volume**	**Marker**	**Allele 1**	**Allele 2**	**Allele 2 AF**	**European ancestry cohort (*****n*** = **208)**		
					***P*** **value**	β	**SE**
Amygdala	rs11111293	C	T	0.808	0.094	−0.077	0.046
Hippocampus	rs77956314	C	T	0.916	0.340	0.046	0.048
*Hippocampus*	*rs61921502*	*G*	*T*	*0.839*	*0.026*	–*0.106*	*0.047*
*Brainstem*	*rs11111090*	*C*	*A*	*0.517*	*0.043*	–*0.072*	*0.036*
Caudate	rs3133370	C	T	0.637	0.605	−0.026	0.050
**Putamen**	**rs945270**	**C**	**G**	**0.423**	**3.67** **×10**^**−3**^	**0.128**	**0.0434**
Pallidum	rs2923447	G	T	0.555	0.373	0.042	0.047
Thalamus	rs12600720	G	C	0.647	0.622	−0.016	0.032

Results of association analyses of the eight SNP-volume pairs in our cohort of 208 European-ancestry neonates. Allele 1 is the coded allele; Allele 2 is the non-coded allele. Standardized beta coefficients (β) and standard errors (SE) are given with respect to Allele 1. The table provides raw *P* values. Results surviving Bonferroni-correction (*P* < 0.00625) are indicated in bold. Results indicating nominal significance (*P* < 0.05) are indicated in italics.

We investigated the SNP-volume associations for the putamen, hippocampus and brainstem in our larger, European-South Asian ancestry and full mixed-ancestry cohorts. The putamen result was also significant in both the European-South Asian ancestry (β = 0.125, SE = 0.040, *P* = 1.89 × 10^−3^) and full mixed-ancestry cohorts (β = 0.122, SE = 0.034, *P* = 4.16 × 10^−4^) suggesting SNP rs945270 may be associated with early putamen development across multiple ancestral groups.

The SNP rs61921502-hippocampal association had an uncorrected *P*-value < 0.05 in both the European-South Asian ancestry (β = −0.101, SE = 0.043, *P* = 0.020) and full mixed-ancestry cohorts (β = −0.071, SE = 0.034, *P* = 0.038), suggesting the association may extend across different ancestries. In contrast, whilst the rs11111090-brainstem association had an uncorrected *P*-value < 0.05 in the European-South Asian ancestry cohort (β = −0.071, SE = 0.032, *P* = 0.029), it was not significant in our full mixed-ancestry cohort (*P* = 0.130). The dilution of the SNP – phenotype relationship in ancestrally mixed populations is well documented (Medina-Gomez et al., 2015) and may be a result of differential tagging in different populations.

For completeness, the additional, non-significant associations were explored in the European-South Asian ancestry and full mixed-ancestry cohorts with results detailed in the Supplementary Tables 2a, b, respectively.

### 3.2 GPSs for adult subcortical brain volume and neonatal brain volume

**Figure 1:**
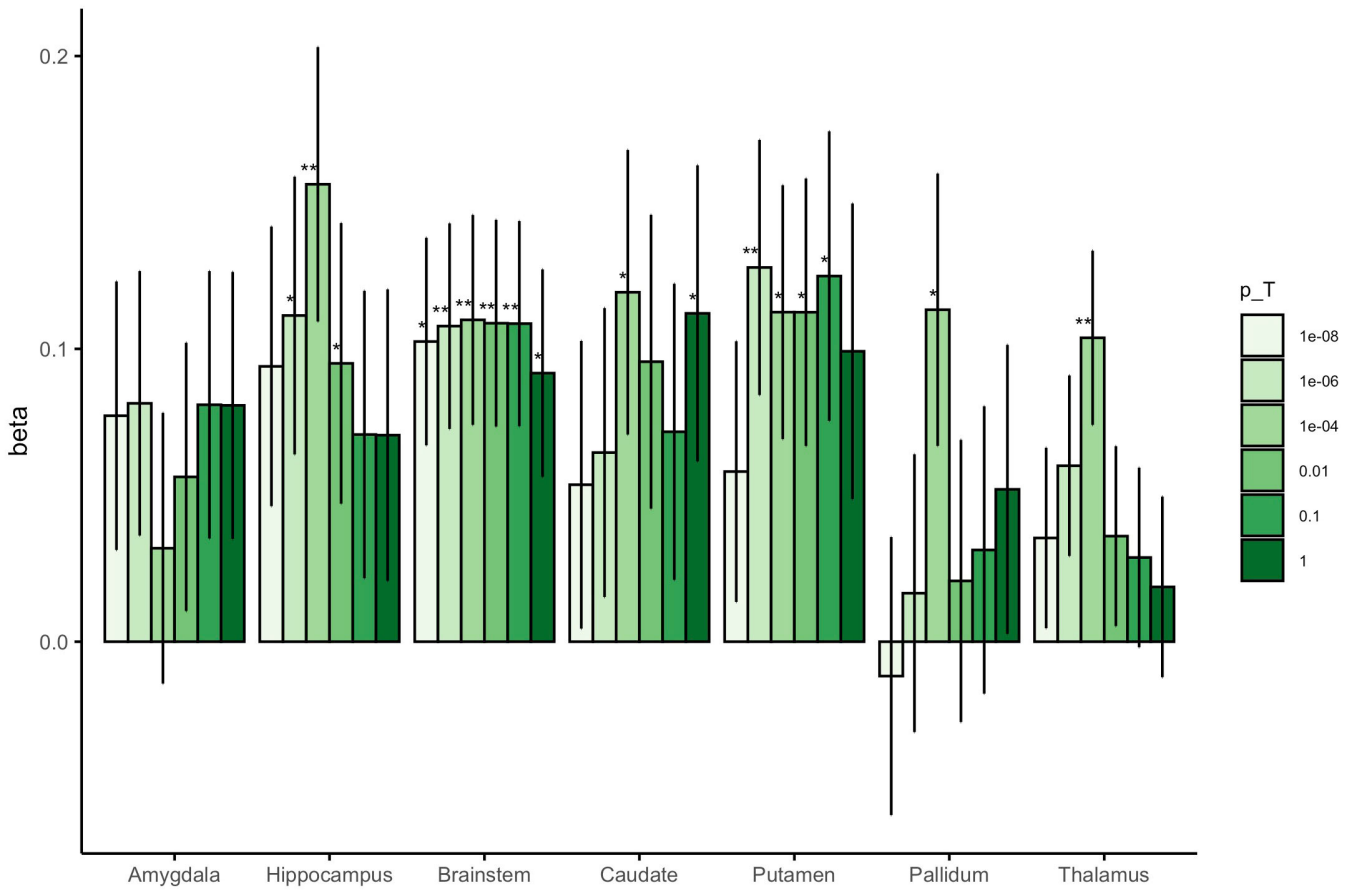
The beta coefficients for the association between adult subcortical GPS and the corresponding neonatal subcortical brain region. The colored bars represent the different values for the GWAS *P*-value thresholds investigated for each GPS (*p*_*T*_ = 1 × 10^−8^, 1 × 10^−6^, 0.0001, 0.01, 0.1 and 1). The error bars indicate standard errors in the beta coefficients. ^**^Results surviving multiple testing correction (*P* < 4.17 × 10^−3^), ^*^Nominal association (uncorrected *P* < 0.05).

Associations between the region-specific GPSs and neonatal brain volumes are shown in Figure 1. We observed a significant association between adult GPS for subcortical volume and infant subcortical volume for the hippocampus, brainstem, putamen, and thalamus (*P* < 4.17 × 10^−3^). These associations were generally seen at a single GPS threshold with other thresholds indicating an uncorrected association or no association. Neonatal brainstem volume was most robustly predicted by adult GPS; neonatal brainstem volume was significantly associated with adult brainstem GPS at four of the six *P-*value thresholds explored.

For completeness, an investigation of non-region-specific associations was also undertaken, the results of which are presented in a series of heatmaps in Supplementary Figure 2. As expected, the most robust associations are the region-specific associations. Adult subcortical GPSs occasionally show uncorrected association with non-region-specific neonatal brain volumes.

## 4 Discussion

This work asks if common genetic variation, known to be important for adult subcortical brain volume, is important in fetal and perinatal brain development. GWAS have explored SNP-phenotype associations for multiple brain-imaging phenotypes. Here we focused on subcortical brain volumes which have been studied in large adult cohorts (Satizabal et al., 2019; Hibar et al., 2017) and for which comparable phenotypes could be extracted in our neonatal cohort.

We explored eight SNP-volume associations that have been established in adult cohorts. Of these, only the association of rs945270 with putamen volume was significant in our neonatal cohort. This is notably the most significant result identified in adult studies (Satizabal et al., 2019; Hibar et al., 2017). rs945270 is an intergenic locus downstream of the *Kinectin 1* (*KTN1*) gene and is associated with altered expression of *KTN1* in both blood and brain tissue (Li et al., 2005). Specifically, Hibar et al. (2015) showed that the C allele, which is associated with larger putamen volume, leads to increased expression of *KTN1* in the frontal cortex and putamen. Two further SNP-volume pairs had uncorrected *P*-values < 0.05 but did not survive multiple-testing correction: SNP rs61921502 with hippocampal volume and SNP rs11111090 with brainstem volume.

In a similar, but significantly larger study than ours, Le Grand et al. (2022), compared genomic loci associated with subcortical volumes in middle-aged and older adults with those associated with the same volumes in younger adults (18–35 years). They looked at 38 genome-wide significant loci and found 10 SNPs significantly associated with same subcortical volume in younger and middle-to-older adults. Interestingly, their most significant association was for a SNP near the gene *KTN1* with putamen volume, which is in strong linkage disequilibrium (LD *r*^2^ = 0.976, D' = 1) with rs945270, the significant SNP-volume result in our neonatal cohort. Several studies have now shown association of genetic variants in this region with putamen volume (Hibar et al., 2015; Satizabal et al., 2019; Le Grand et al., 2022) and our results, which replicate this association at birth, provide evidence for an important role of this locus in shaping putamen structure across the life-course.

Our dHCP cohort includes infants of diverse ancestry. This diversity, an asset of this cohort, presents challenges in imaging-genomic studies. The GWAS results we explored, and the summary statistics used to compute the GPSs were from predominantly European-ancestry individuals. For this reason, our main analysis was undertaken in a European-ancestry subsample of the dHCP. However, for the single SNP-volume investigations, we were interested to know if the results from the European cohort were generalizable to a larger, ancestrally diverse cohort.

We explored our significant and nominally significant SNP-volume associations in both our European-South Asian ancestry and full mixed-ancestry cohorts. The rs945270-putamen association was present across all cohorts and was more robust in our larger European-South Asian ancestry and full mixed-ancestry cohorts. This is consistent with the results of Satizabal et al. (2019) who found the direction of the rs945270 – putamen association was the same for all ancestries and the strength of association increased in a meta-analysis including different populations.

The results for rs61921502 and rs11111090, which had uncorrected *P*-values < 0.05 in our European-ancestry cohort, but did not survive multiple-testing correction, were more variable. The SNP rs61921502 - hippocampal association retained an uncorrected *P*-value < 0.05 in our European-South Asian ancestry and full mixed-ancestry cohorts but the association was not more robust in these larger groups. In adult studies, Hibar et al. (2017) found SNP rs61921502 was borderline associated with hippocampal volume in an African cohort but found no association in the Asian or Mexican-American ancestry cohorts. The SNP rs11111090-brainstem association also had an uncorrected *P*-value < 0.05 in our European-South Asian ancestry cohort but showed no association in the full mixed-ancestry cohort. The adult GWAS did not see concordance in the direction of association of SNP rs11111090 with brainstem volume for different ancestries and the meta-analysis including non-European ancestry individuals was less significant than in the European-only ancestry group (Satizabal et al., 2019). The heterogeneity of effect sizes in more ancestrally diverse cohorts is expected. The causal variant may be consistent across the different ancestral groups however differences in the LD between different cohorts will affect how well the studied SNP tags the true causal variant (Peterson et al., 2019).

Work in the literature has started to explore the role of genetic variation in early brain development. However, the large sample sizes required for hypothesis-free GWAS makes such studies of brain-imaging phenotypes in infants scarce. Xia et al. (2017) undertook a GWAS of global brain tissue volumes in 561 infants and compared their results with two large-scale neuroimaging GWAS, one in adolescents and another in adults. They found only minimal overlap between common variants impacting brain volume at different ages. In contrast, in a large, but predominantly adult study that undertook GWAS for seven subcortical brain volumes, Hibar et al. (2015) asked if the phenotypic effects of SNPs were related to the age of individuals in the GWAS. Only one of the eight significant loci they identified appeared to have an age-related effect and they argued that most SNP-volume effects, including the rs945270-putamen association, are likely to be stable across the life course. Nonetheless, their cohort did not include infants or very young children.

Complementary to our investigation of specific genetic loci we also computed GPSs for adult subcortical brain volumes in our neonatal cohort and asked if they were associated with the corresponding brain volume at birth. We found significant associations between GPSs for adult hippocampal, brainstem, putamen and thalamic volumes and the corresponding neonatal volume.

In the supplementary analysis we present results for non-region-specific associations for the subcortical GPSs and neonatal brain volumes. We observed three cross-region associations: between hippocampal GPS and amygdala volume, brainstem GPS and hippocampal volume, and caudate GPS and putamen volume. Prior studies have demonstrated significant genetic overlap between subcortical volumes in adults (Satizabal et al., 2019; Le Grand et al., 2022) with robust associations reported for caudate-putamen and hippocampal-amygdala volumes, consistent with two of the three associations observed in our neonatal cohort. The brainstem-hippocampal association has not been previously reported in adult cohorts. Future studies with larger neonatal cohorts will be essential to determine the reproducibility and biological significance of these associations.

Our work adds to the emerging literature exploring the role of genetic polymorphisms, known to be important in shaping adult brain morphology, in early brain development. We compare our results to those of Lamballais et al. (2021) who computed GPSs for adult subcortical brain volumes and asked how predictive they are of pediatric subcortical brain volume and infant gangliothalamic ovoid diameter (assessed via ultrasound). In their pediatric cohort, imaged at around 10 years, they observed robust associations between all adult GPS scores and the corresponding pediatric subcortical brain volumes. Interestingly, both our study and theirs find the strongest associations for the putamen and brainstem; genetic polymorphisms clearly exist that are important in shaping the morphology of these structures from fetal life through childhood to adulthood.

In general, our subcortical GPS-volume results imply weaker associations than the pediatric sample of Lamballais et al. (2021). This may be a result of our smaller cohort but is also likely influenced by our earlier imaging timepoint. The pediatric brain reaches its maximum size between 10 and 12 years (Giedd, 2004) and we know the subcortical brain develops rapidly during early childhood (Dima et al., 2021). Our neonatal cohort represents a snapshot in the perinatal period soon after birth and fetal development. It is possible that genetic variation plays a less measurable role in shaping the subcortical brain during fetal and perinatal development as compared with later in childhood. It is also possible that the genes and gene pathways most important in influencing subcortical morphology at birth may differ from those in older children and adults.

Studies exploring heritability of brain imaging phenotypes over the life course suggest that it may increase slightly from infancy to childhood (Gilmore et al., 2010; Jansen et al., 2015). Furthermore, work looking at genetic variation important in the rate of change of brain structure suggests that heritability is higher in adults than in children implying genetic variation may play a greater role in explaining structural changes as individuals get older. The notion that there may be other important factors relevant early in the life-course is supported by the mediation analysis of Lamballais et al. (2021) which suggests that the genetic effects on subcortical volumes during infancy explain only a small part of the volumetric variation seen in early childhood.

A significant limitation of this work is our small sample size. This study was undertaken in a substantially smaller cohort than the adult and pediatric studies with which we compare our results (Lamballais et al., 2021; Alemany et al., 2019). The primary adult studies used for SNP-volume exploration and from which our GPS for subcortical volumes were computed (Satizabal et al., 2019; Hibar et al., 2017) included upwards of 30,000 individuals. Despite our limited numbers, we observe the rs945270-putamen association that has also been replicated in a young adult cohort (Le Grand et al., 2022), showing for the first time that this association exists from birth. Further, we find that GPSs for adult brainstem, hippocampal, putamen and thalamic volumes are all significantly associated with the corresponding neonatal subcortical volumes. Given the limitations of our sample size, the results presented here should be considered a starting point in better understanding genomic variation important in shaping neonatal brain volume. We hope that similar work will be undertaken in independent neonatal samples as they become available.

## 5 Conclusions

Our results indicate that the common genetic variation important for adult subcortical brain volume plays a significant role in fetal and perinatal brain development. They also suggest that the genetic variability most relevant in shaping neonatal brain morphology may not be fully represented by the genetic variability shaping adult brain morphology. It is possible that during fetal and perinatal development, other factors, including genes and genetic pathways distinct to this period, may play prominent roles. Further analysis with fetal and neonatal cohorts is required to better understand how the genetic mechanisms shaping brain morphology might differ in this early developmental period.

## Data Availability

Publicly available datasets were analyzed in this study. This data can be found at: https://data.developingconnectome.org/ and https://nda.nih.gov/edit_collection.html?id=3955.

## References

[B1] Alemany S. Jansen P. R. Muetzel R. L. Marques N. El Marroun H. Jaddoe V. W. V.. (2019). Common polygenic variations for psychiatric disorders and cognition in relation to brain morphology in the general pediatric population. J. Am. Acad. Child Adolesc. Psychiatry. 58, 600–607. 10.1016/j.jaac.2018.09.443 30768412

[B2] Auton A. Abecasis G. R. Altshuler D. M. Durbin R. M. Bentley D. R. Chakravarti A.. (2015). A global reference for human genetic variation. Nature 526, 68–74. 10.1038/nature15393 26432245 PMC4750478

[B3] Boardman J. P. Counsell S. J. Rueckert D. Kapellou O. Bhatia K. K. Aljabar P.. (2006). Abnormal deep grey matter development following preterm birth detected using deformation-based morphometry. Neuroimage 32, 70–78. 10.1016/j.neuroimage.2006.03.029 16675269

[B4] Boardman J. P. Craven C. Valappil S. Counsell S. J. Dyet L. E. Rueckert D.. (2010). A common neonatal image phenotype predicts adverse neurodevelopmental outcome in children born preterm. Neuroimage 52, 409–414. 10.1016/j.neuroimage.2010.04.261 20451627

[B5] Choi S. W. O'Reilly P. F. (2019). PRSice-2: polygenic risk score software for biobank-scale data. Gigascience 8, 1–6. 10.1093/gigascience/giz082 31307061 PMC6629542

[B6] Cordero-Grande L. Hughes E. J. Hutter J. Price A. N. Hajnal J. V. (2018). Three-dimensional motion corrected sensitivity encoding reconstruction for multi-shot multi-slice MRI: application to neonatal brain imaging. Magn. Reson. Med. 79, 1365–1376. 10.1002/mrm.26796 28626962 PMC5811842

[B7] Dima D. Modabbernia A. Papachristou E. Doucet G. E. Agartz I. Aghajani M.. (2021). Subcortical volumes across the lifespan: data from 18,605 healthy individuals aged 3–90 years. Hum. Brain Mapp. 452–69. 10.1002/hbm.25320 33570244 PMC8675429

[B8] Edwards A. D. Rueckert D. Smith S. M. Abo Seada S. Alansary A. Almalbis J.. (2022). The developing human connectome project neonatal data release. Front. Neurosci. 16, 1–14. 10.3389/fnins.2022.886772 35677357 PMC9169090

[B9] Elliott L. T. Sharp K. Alfaro-almagro F. Shi S. Miller K. L. Douaud G.. (2018). Genome-wide association studies of brain imaging phenotypes in UK Biobank. Nature. 562, 210–216. 10.1038/s41586-018-0571-7 30305740 PMC6786974

[B10] Ellison-Wright I. Ellison-Wright Z. Bullmore E. (2008). Structural brain change in attention deficit hyperactivity disorder identified by meta-analysis. BMC Psychiatry. 8, 1–8. 10.1186/1471-244X-8-51 18590567 PMC2453122

[B11] Giedd J. N. (2004). Structural magnetic resonance imaging of the adolescent brain. Ann. N. Y. Acad. Sci. 1021, 77–85. 10.1196/annals.1308.009 15251877

[B12] Gilmore J. H. Schmitt J. E. Knickmeyer R. C. Smith J. K. Lin W. Styner M.. (2010). Genetic and environmental contributions to neonatal brain structure: a twin study. Hum Brain Mapp. 31, 1174–1182. 10.1002/hbm.20926 20063301 PMC3109622

[B13] Gousias I. S. Edwards A. D. Rutherford M. A. Counsell S. J. Hajnal J. V. Rueckert D.. (2012). Magnetic resonance imaging of the newborn brain: manual segmentation of labelled atlases in term-born and preterm infants. Neuroimage 62, 1499–1509. 10.1016/j.neuroimage.2012.05.083 22713673

[B14] Hibar D. P. Adams H. H. H. Jahanshad N. Chauhan G. Stein J. L. Hofer E.. (2017). Novel genetic loci associated with hippocampal volume. Nat. Commun. 8, 13624. 10.1038/ncomms13624 28098162 PMC5253632

[B15] Hibar D. P. Stein J. L. Renteria M. E. Arias-Vasquez A. Desrivières S. Jahanshad N.. (2015). Common genetic variants influence human subcortical brain structures. Nature 520, 224–229. 10.1038/nature14101 25607358 PMC4393366

[B16] Hughes E. J. Winchman T. Padormo F. Teixeira R. Wurie J. Sharma M.. (2017). A dedicated neonatal brain imaging system. Magn. Reson. Med. 78, 794–804. 10.1002/mrm.26462 27643791 PMC5516134

[B17] Inder T. E. Warfield S. K. Wang H. Hu P. S. (2005). Abnormal cerebral structure is present at term in premature infants. Pediatrics 115, 286–294. 10.1542/peds.2004-0326 15687434

[B18] Jansen A. G. Mous S. E. White T. Posthuma D. Polderman T. J. C. (2015). What twin studies tell us about the heritability of brain development, morphology, and function: a review. Neuropsychol. Rev. 25, 27–46. 10.1007/s11065-015-9278-9 25672928 PMC4412550

[B19] Kuklisova-Murgasova M. Quaghebeur G. Rutherford M. A. Hajnal J. V. Schnabel J. A. (2012). Reconstruction of fetal brain MRI with intensity matching and complete outlier removal. Med. Image Anal. 16, 1550–1564. 10.1016/j.media.2012.07.004 22939612 PMC4067058

[B20] Lamballais S. Jansen P. R. Labrecque J. A. Ikram M. A. White T. (2021). Genetic scores for adult subcortical volumes associate with subcortical volumes during infancy and childhood. Hum. Brain Mapp. 42, 1583–1593. 10.1002/hbm.25292 33528897 PMC7978120

[B21] Le Grand Q. Satizabal C. L. Sargurupremraj M. Mishra A. Soumar,é A. Laurent A.. (2022). Genomic studies across the lifespan point to early mechanisms determining subcortical volumes. Biol. Psychiatry Cogn. Neurosci. Neuroimaging. 7, 616–628. 10.1016/j.bpsc.2021.10.011 34700051 PMC9395126

[B22] Li J. Adjusting J. i. L. (2005). multiple testing in multilocus analyses using the eigenvalues of a correlation matrix. Heredity 95, 221–227. 10.1038/sj.hdy.6800717 16077740

[B23] Makropoulos A. Gousias I. S. Ledig C. Aljabar P. Serag A. Hajnal J. V.. (2014). Automatic whole brain MRI segmentation of the developing neonatal brain. IEEE Trans. Med. Imaging 33, 1818–1831. 10.1109/TMI.2014.2322280 24816548

[B24] Makropoulos A. Robinson E. C. Schuh A. Wright R. Fitzgibbon S. Bozek J.. (2018). The developing human connectome project: a minimal processing pipeline for neonatal cortical surface reconstruction. Neuroimage 173, 88–112. 10.1016/j.neuroimage.2018.01.054 29409960 PMC6783314

[B25] McCarthy S. Das S. Kretzschmar W. Delaneau O. Wood A. R. Teumer A.. (2016). A reference panel of 64,976 haplotypes for genotype imputation. Nat. Genet. 48, 1279–1283. 10.1038/ng.3643 27548312 PMC5388176

[B26] Medina-Gomez C. Felix J. F. Estrada K. Peters M. J. Herrera L. Kruithof C. J.. (2015). Challenges in conducting genome-wide association studies in highly admixed multi-ethnic populations: the generation R study. Eur. J. Epidemiol. 30, 317–330. 10.1007/s10654-015-9998-4 25762173 PMC4385148

[B27] Patel H. Lee S. H. Breen G. Menzel S. Ojewunmi O. Dobson R. J. B.. (2022). The COPILOT raw illumina genotyping QC protocol. Curr. Protoc. 2, 1–30. 10.1002/cpz1.373 35452565

[B28] Peterson R. E. Kuchenbaecker K. Walters R. K. Chen C. Y. Popejoy A. B. Periyasamy S.. (2019). Genome-wide association studies in ancestrally diverse populations: opportunities, methods, pitfalls, and recommendations. Cell. 179, 589–603. 10.1016/j.cell.2019.08.051 31607513 PMC6939869

[B29] Satizabal C. L. Adams H. H. H. Hibar D. P. White C. C. Knol M. J. Stein J. L.. (2019). Genetic architecture of subcortical brain structures in 38,851 individuals. Nat. Genet. 51, 1624–1636. 10.1038/s41588-019-0511-y 31636452 PMC7055269

[B30] Schmaal L. Veltman D. J. van Erp T. G. M. Sämann P. G. Frodl T. Jahanshad N.. (2016). Subcortical brain alterations in major depressive disorder: findings from the ENIGMA major depressive disorder working group. Mol Psychiatry. 21, 806–812. 26122586 10.1038/mp.2015.69 PMC4879183

[B31] Schuh A. Makropoulos A. Robinson E. C. Cordero-Grande L. Hughes E. Hutter J.. (2018). Unbiased construction of a temporally consistent morphological atlas of neonatal brain development. bioRxiv [Preprint]. 10.1101/251512

[B32] Shepherd G. M. G. (2013). Corticostriatal connectivity and its role in disease. Nat. Rev. Neurosci. 14, 278–291. 10.1038/nrn3469 23511908 PMC4096337

[B33] Shi F. Yap P. T. Wu G. Jia H. Gilmore J. H. Lin W.. (2011). Infant brain atlases from neonates to 1- and 2-year-olds. PLoS ONE 6, e18746. 10.1371/journal.pone.0018746 21533194 PMC3077403

[B34] Silbereis J. C. Pochareddy S. Zhu Y. Li M. Sestan N. (2016). The cellular and molecular landscapes of the developing human central nervous system. Neuron 89, 248. 10.1016/j.neuron.2015.12.008 26796689 PMC4959909

[B35] Srinivasan L. Dutta R. Counsell S. J. Allsop J. M. Boardman J. P. (2007). Quantification of Deep gray matter in preterm infants at term-equivalent age using manual volumetry of 3-tesla magnetic resonance images. Pediatrics 119, 759–765. 10.1542/peds.2006-2508 17403847

[B36] Taoudi-Benchekroun Y. Christiaens D. Grigorescu I. Gale-Grant O. Schuh A. Pietsch M.. (2022). Predicting age and clinical risk from the neonatal connectome. Neuroimage. 257, 119319. 10.1016/j.neuroimage.2022.119319 35589001

[B37] Van Erp T. G. M. Hibar D. P. Rasmussen J. M. Glahn D. C. Pearlson G. D. Andreassen O. A.. (2016). Subcortical brain volume abnormalities in 2028 individuals with schizophrenia and 2540 healthy controls via the ENIGMA consortium. Mol Psychiatry. 21, 547–553. 10.1038/mp.2015.63 26033243 PMC4668237

[B38] Xia K. Zhang J. Ahn M. Jha S. Crowley J. J. Szatkiewicz J.. (2017). Genome-wide association analysis identifies common variants influencing infant brain volumes. Transl Psychiatry. 7, 1–10. 10.1038/tp.2017.159 28763065 PMC5611727

