# Supplementary material for: Common genetic variation associated with adult subcortical brain volume is also associated with subcortical brain volume at birth
Source: Front Neurosci. 2025 Jun 10;19:1546845. doi: 10.3389/fnins.2025.1546845 (PMC12185412; doi:10.3389/fnins.2025.1546845)
Supplement: Supplementary file 1 [file Data_Sheet_1.docx]

Supplementary Material

**Common Genetic Variation Associated with Adult Subcortical Brain Volume is also Associated with Subcortical Brain Volume at Birth**

Authors: Harriet Cullen^1,2^, Konstantina Dimitrakopoulou^3^, Hamel Patel^4^, Charles Curtis^4^, Dafnis Batalle^1,5^, Oliver Gale-Grant^1,5^, Lucilio Cordero-Grande^1,6^, Anthony Price^1^, Joseph V. Hajnal^1^, A. David Edwards^1^

*^1^Centre for the Developing Brain, School of Biomedical Engineering and Imaging Sciences, King’s College, London, UK,*

*^2^Department of Medical and Molecular Genetics, School of Basic and Medical Biosciences, King’s College London, UK*

*^3^Translational Bioinformatics Platform, NIHR Biomedical Research Centre, Guy's and St Thomas' NHS Foundation Trust and King's College London, UK,*

*^4^NIHR BioResource Centre Maudsley, NIHR Maudsley Biomedical Research Centre, King's College London, UK,*

*^5^ Department of Forensic and Neurodevelopmental Sciences, Institute of Psychiatry, Psychology & Neuroscience, King's College London, UK*

*^6^ Biomedical Image Technologies, ETSI Telecomunicación, Universidad Politécnica de Madrid & CIBER-BBN, ISCIII, Madrid, Spain*

**Corresponding author:** Harriet Cullen

**Email:** harriet.cullen@kcl.ac.uk

**This file includes:**

**Supplementary Tables**: Supplementary Tables ST1, ST2a and ST2b.

**Supplementary Figures:** Supplementary Figures SF1 and SF2

**Supplementary References**

**Supplementary Table ST1:** SNP-volume pairs considered for exploration in our neonatal cohort.

| **Subcortical Brain Volume** | **SNP** | **Chromosome** | **Position** | **Allele 1/ Allele 2** | **Location** | **Original adult study *P* value** (1) (2) |
| --- | --- | --- | --- | --- | --- | --- |
| Amygdala | ﻿rs11111293 | 12 | 102,921,296 | T/C | Intergenic | ﻿4.16 × 10^−10^ |
| Hippocampus | rs77956314 | 12 | 117,323,367 | T/C | Intergenic | 2.06 × 10^−25^ |
| Hippocampus | rs61921502 | 12 | 65,832,468 | T/G | Intronic | 1.94 × 10^−19^ |
| Brainstem | ﻿rs11111090 | 12 | 102,326,461 | A/C | Intergenic | ﻿3.70 × 10^−27^ |
| Caudate nucleus | ﻿rs3133370 | 11 | 92,026,446 | T/C | Intergenic | ﻿5.59 × 10^−14^ |
| Putamen | ﻿rs945270 | 14 | 56,200,473 | C/G | Intergenic | ﻿5.02 × 10^−51^ |
| Globus pallidus | ﻿rs2923447 | 8 | 42,439,848 | T/G | Intergenic | ﻿4.88 × 10^−16^ |
| Thalamus | ﻿rs12600720 | 17 | 78,448,640 | C/G | Intronic | ﻿4.06 × 10^−10^ |

**Supplementary Table ST1**. ﻿Information on the eight SNPs most robustly associated with subcortical brain volumes from studies (1) and (2). The table details the SNP, chromosome and base pair position (GRCh37), allele 1 and 2 for the SNP in question, the SNP function and the P value of association in the original adult study. The nucleus accumbens is not included because this volume was not extracted using our neonatal atlases.

**Supplementary Table ST2a**: SNP-volume associations showing results for the European (n=208), European-South Asian (n=258), and full mixed-ancestry cohorts (n=418).

| Volume | Marker and allele 1/2 | European ancestry cohort (n=208) | | European-South Asian ancestry cohort (n=258) | | Full mixed-ancestry cohort (n=418) | |
| --- | --- | --- | --- | --- | --- | --- | --- |
|  |  | *P* value | β ± SE | *P* value | β ± SE | *P* value | β ± SE |
| Amygdala | rs11111293 (C/T) | 0.094 | -0.077 ± 0.046 | *0.044* | *-0.083 ± 0.041* | 0.053 | -0.063 ± 0.033 |
| Hippocampus | rs77956314 (C/T) | 0.340 | 0.046 ± 0.048 | 0.168 | 0.060 ± 0.044 | 0.230 | 0.041 ± 0.034 |
| Hippocampus | rs61921502 (G/C) | *0.026* | *-0.106 ± 0.047* | *0.020* | *-0.101 ± 0.043* | *0.038* | *-0.071 ± 0.034* |
| Brainstem | rs11111090 (C/A) | *0.043* | *-0.072 ± 0.036* | *0.029* | *-0.071 ± 0.032* | 0.130 | -0.043 ± 0.028 |
| Caudate | rs3133370 (C/T) | 0.605 | -0.026 ± 0.050 | 0.278 | -0.049 ± 0.045 | 0.594 | -0.019 ± 0.035 |
| Putamen | rs945270 (C/G) | **3.67x10^-3^** | **0.128 ± 0.0434** | **1.89x10^-3^** | **0.125 ± 0.040** | **4.16x10^-4^** | **0.122 ± 0.034** |
| Pallidum | rs2923447 (G/T) | 0.373 | 0.042 ± 0.047 | 0.496 | 0.030 ± 0.043 | 0.449 | 0.025 ± 0.033 |
| Thalamus | rs12600720 (G/C) | 0.622 | -0.016 ± 0.032 | 0.765 | -0.009 ± 0.031 | 0.803 | 0.008 ± 0.032 |

**Supplementary Table ST2a.** ﻿Results of association analyses for the eight selected SNP-volume pairs in our European, European-South Asian, and full mixed-ancestry neonatal cohorts. Allele 1 is the coded allele; Allele 2 is the non-coded allele. Standardized beta coefficients (β) and standard errors (SE) are given with respect to Allele 1. The table provides raw *P* values. Results surviving Bonferroni-correction (*P* <0.0083) are indicated in bold. Results indicating nominal significance (*P* <0.05) are indicated in italics.

**Supplementary Table ST2b**: Allele frequencies in the European (n=208), European-South Asian (n=258), and full mixed-ancestry cohorts (n=418).

| Volume | Marker | Allele 1 | Allele 2 | Allele 2 AF | | |
| --- | --- | --- | --- | --- | --- | --- |
|  |  |  |  | European | European – South Asian | Mixed ancestry |
| Amygdala | rs11111293 | C | T | 0.808 | 0.804 | 0.851 |
| Hippocampus | rs77956314 | C | T | 0.916 | 0.918 | 0.920 |
| Hippocampus | rs61921502 | G | T | 0.839 | 0.857 | 0.889 |
| Brainstem | rs11111090 | C | A | 0.517 | 0.537 | 0.627 |
| Caudate | rs3133370 | C | T | 0.637 | 0.653 | 0.633 |
| Putamen | rs945270 | C | G | 0.423 | 0.441 | 0.537 |
| Pallidum | rs2923447 | G | T | 0.555 | 0.566 | 0.585 |
| Thalamus | rs12600720 | G | C | 0.647 | 0.672 | 0.719 |

**Supplementary Table ST2b.** detailing the allele frequency of allele 2 in the European, European-South Asian, and mixed ancestry cohorts respectively. ﻿

**Supplementary Figure 1 (SF1): Flow Chart of Study Populations**

**
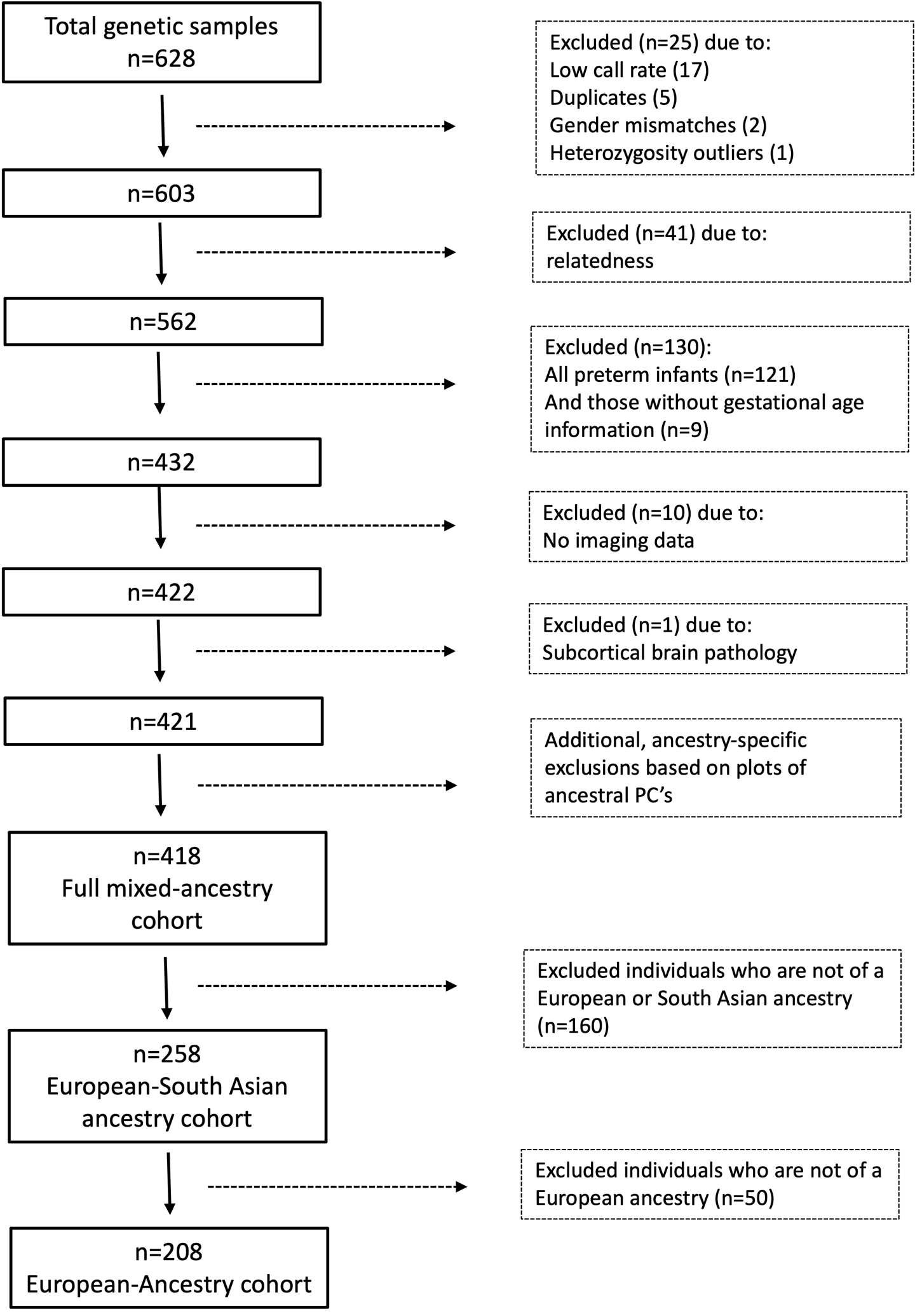
**

**Supplementary Figure 2 (SF2):** Heatmaps for association between adult subcortical genome-wide polygenic scores and neonatal brain volume.**

**

**﻿Supplementary Figure 2 (SF2).** Heatmaps for the associations between GPSs for seven adult subcortical brain volumes and the corresponding neonatal brain volumes. Each heatmap corresponds to the GPSs for a different adult subcortical brain volume: Amygdala (a), Hippocampus (b), Brainstem (c), Caudate (d), Putamen (e), Pallidum (f) and Thalamus (g). The horizontal axis gives the GPS at the six GWAS *P* value thresholds (p_T_ = 1x10^-8^, 1x10^-6^, 0.0001, 0.01, 0.1 and 1) and the vertical axis the seven neonatal subcortical brain volumes. ﻿**P* value < 0.05; ** *P* value <0.01; ***P value < 4.17x10^-3^.

**Supplementary Figure 3 (SF3):** Principal component analysis (PCA) of the dHCP cohort merged with the 1000 Genomes Project population reference samples (AFR: African; AMR: Admixed American; EAS: East Asian; EUR: European; SAD: South Asian).


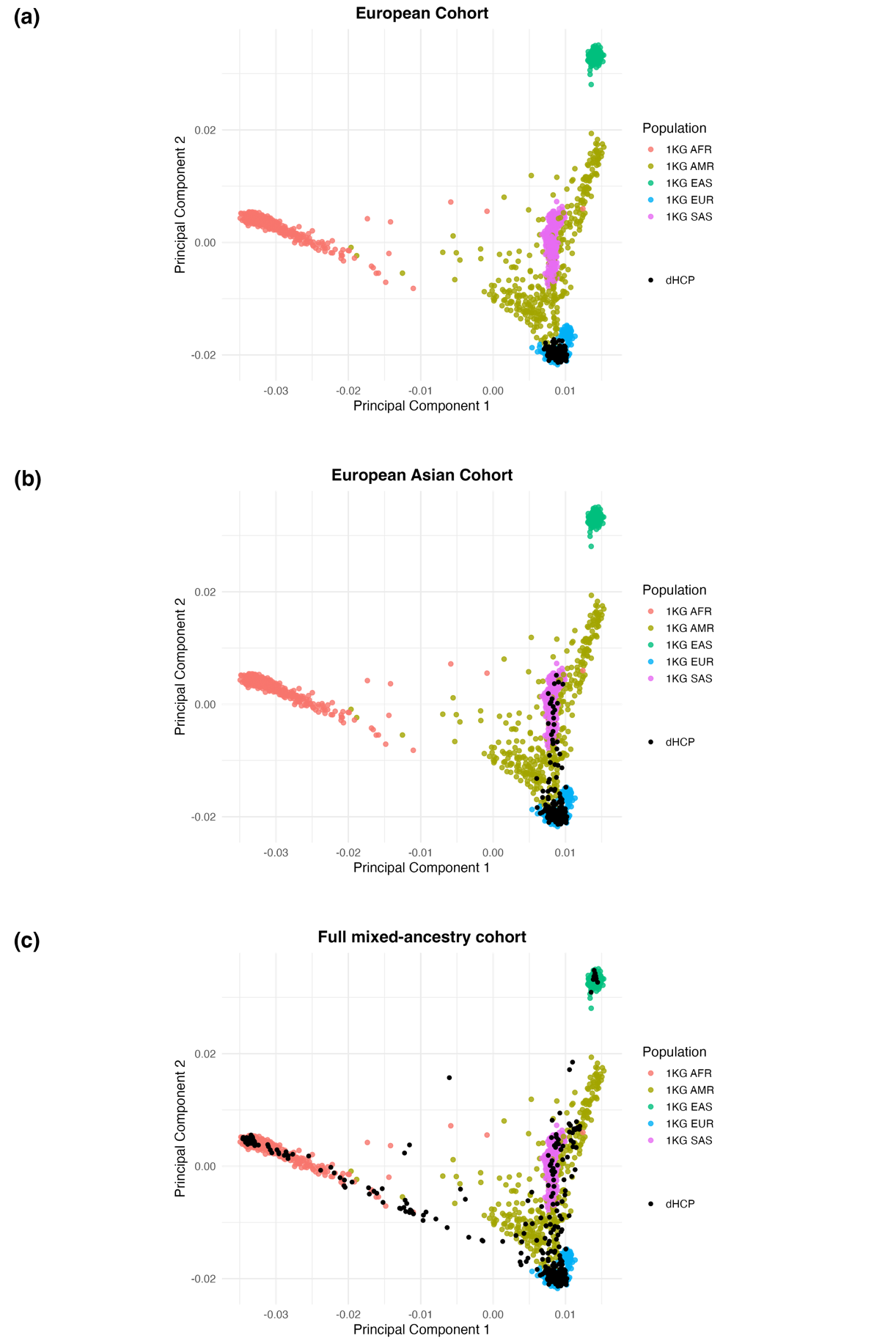


**Supplementary Figure 3 (SF3):** PCA of the dHCP cohort merged with the 1000 Genomes Project population (1KG) reference samples (AFR: African; AMR: Admixed American; EAS: East Asian; EUR: European; SAS: South Asian). Plot (a) shows the European-only cohort which was the principal cohort used in this work (n=208), plot (b) shows the European-South Asian cohort (n=258) and plot (c) shows the full mixed-ancestry cohort.

**Supplementary References**

1. Satizabal CL, Adams HHH, Hibar DP, White CC, Knol MJ, Stein JL, et al. Genetic architecture of subcortical brain structures in 38,851 individuals. Nat Genet [Internet]. 2019;51(November). Available from: http://www.ncbi.nlm.nih.gov/pubmed/31636452

2. Hibar DP, Adams HHH, Jahanshad N, Chauhan G, Stein JL, Hofer E, et al. Novel genetic loci associated with hippocampal volume. Nat Commun. 2017;8.
